# Electromagnetic Thermography Nondestructive Evaluation: Physics-based Modeling and Pattern Mining

**DOI:** 10.1038/srep25480

**Published:** 2016-05-09

**Authors:** Bin Gao, Wai Lok Woo, Gui Yun Tian

**Affiliations:** 1 School of Automation, University of Electronic Science and Technology of China, 610054, Chengdu, China; 2 School of Electrical and Electronic Engineering, Newcastle University, NE1 7RU, England, United Kingdom

## Abstract

Electromagnetic mechanism of Joule heating and thermal conduction on conductive material characterization broadens their scope for implementation in real thermography based Nondestructive testing and evaluation (NDT&E) systems by imparting sensitivity, conformability and allowing fast and imaging detection, which is necessary for efficiency. The issue of automatic material evaluation has not been fully addressed by researchers and it marks a crucial first step to analyzing the structural health of the material, which in turn sheds light on understanding the production of the defects mechanisms. In this study, we bridge the gap between the physics world and mathematical modeling world. We generate physics-mathematical modeling and mining route in the spatial-, time-, frequency-, and sparse-pattern domains. This is a significant step towards realizing the deeper insight in electromagnetic thermography (EMT) and automatic defect identification. This renders the EMT a promising candidate for the highly efficient and yet flexible NDT&E.

Since 1960s, thermal testing has been successfully explored in NDT&E applications^1^,^2^ to measure the surface temperature variations in response to induced energy. The energy generates a temperature contrast at material discontinuities that can be detected by an infrared (IR) camera. It was the development of the infrared camera in the late 1970s which made it possible to directly detect the temperature contrast over large inspection areas. The IR cameras detect radiation in the IR range of the electromagnetic spectrum and generate images of IR or thermal emission called thermograms, allowing very sensitive non-contact temperature measurement. Thermal testing is also used for defect characterization and material property evaluation and inspection since it is completely noncontact and offers for the rapid inspection over a large area within a short time. It can be used in a wide range of areas^3^, such as in agriculture, civil engineering and architecture, diagnosing electrical equipments, automotive industry, medicine and biology, manufacturing industry, food quality control and protection of historic heritage. Thermal testing is generally divided into two main streams: passive infrared thermography (PIT) and active infrared thermography (AIT). Passive Thermography (PIT) is defined as measuring the temperature differences between the target materials and the surroundings under different ambient temperature conditions. AIT^4^ was developed to provide more accurate information by considering the amount of thermal radiation and heat transfer. The common thermal stimulation techniques in AIT^5^ are: pulsed thermography (e.g., flash thermography), step heating (long pulse), lock-in thermography, and vibrothermography (e.g., ultrasonic IR thermography). Traditional thermal radiation heating is the earliest and the simplest direct technique in AIT. The method employs a lighting source or radiative source to heat the surface of the test object. Flaws or suspicious response can be captured, according to the slow heat transfer, by an infrared (IR) camera inspecting system.

Electromagnetic thermography (EMT)^6^,^7^,^8^,^9^,^10^,^11^,^12^,^13^,^14^,^15^ which combines eddy current (EC), magnetic and thermography, and involves the application for a short period of a high current electromagnetic pulse to the conductive material under inspection. In comparison with other thermography NDT&E techniques, the heat in EMT is not limited to the sample surface, rather it can reach a certain depth, which governed by the skin depth of eddy current. Furthermore, EMT focuses the heat on the defect due to friction or eddy current distortion, and subsequently increase the temperature contrast between the defective region and defect-free areas. From point of view of inspection depth, flash thermography is adaptable by changing the lock-in frequency for defects at different depths. From adaptability in terms of defect orientation, EMT can enhance specific excitation direction to optimize the directional evaluation along the defect orientation which is more effective for geometrically complex components and showed more crack indication. Furthermore, EMT allows area imaging of defects without scanning and enables the detection of both magnetic and non-magnetic metals with rich frequency components fabricated from excitation power. In addition, for optic-excited thermography, the disadvantage also includes the duration until the heat response of the crack reaches the surface is twice longer than with EMT testing. Since the test part is passing through the testing plant and the thermal wave just has a very short time to go through the material, this is not a practical testing method. EMT can only work with conductive material and it has already been applied in small defects detection for compressor blades^16^, impact damage and delamination detection of composite^17^,^18^, probability of detection (POD) estimation^10^ of fatigue cracks and multiple cracks detection.

All above works require signal processing tools to enable defects or materials characterization analysis. Running temperature contrast method^19^ uses the source temperature image which is replaced with the image of the defective thermal image divided by the non-defective thermal image where the process is independent of heating power. However, this method requires manually selection and it may enhance random noise as well. Differentiated contrast method^20^ assumes that at earlier times, any sample behaves as a semi-infinite body where no human selection is necessary. It is only applicable for shallow defects and works for a sample that can be considered as a semi-infinite body. Deviation from these conditions rendered the method ineffective. Polynomial fitting method^21^ attempts to fit noisy temperature signals with polynomial functions. IR image sequence of arbitrary length can be replaced with images of a few polynomial coefficients; however, the images of polynomial coefficients lose the physics of heat conduction. Neural networks^22^ are proposed as universal technique that takes into consideration subtle variations in signal evolution. Each class of test objects requires special training of the network. In thermal testing, several thermal transient response features have been used as an indicator of defect status, which is critical for acceptance/rejection decisions for maintenance and lifetime prediction^23^. To enhance the flaw contrast and improve noise rejection qualities, pattern based image enhancement has been conducted by introducing the raw data upon a set of orthogonal basis functions. Fourier transform has been applied to pulsed thermography, and enhanced the flaw-contrast significantly using phase map^24^ and image normalization^25^. Influence of non-uniform heating and surface emissivity variation was removed by using a Fourier transformation based image reconstruction algorithm^26^. Instead of a prescribed set of basis functions, empirical orthogonal functions were also employed to maximize the anomalous patterns of transient response. The efficiency of principal component analysis (PCA) and independent component analysis (ICA) has been compared on thermography features extraction by considering the initial sequence as either a set of images or a set of temporal profiles^27^. In reviewing the current defect detection methods for IT, two main challenges have been identified. Firstly, the lack of capability in automatically detecting and identifying the defects, and secondly, the need to enhance the detectability and resolution of different types of defects which is the key point to achieve Quantitative NDT&E. However, most recent methods are limited on manually selecting the proper contrast components and pattern extraction based methods are only employed as a signal processing tool. The mathematical justification as to why these algorithms can enhance an image and how these techniques are linked to physics models are not provided. The results are acceptable but generally not predictable. The proper contrast components have to be empirically selected. This ambiguous case prevents the use of thermal testing based NDT&E in automated environments.

From the physics viewpoint, in particular the NDT&E, physics-mathematical pattern modeling and mining (PPMM) has already started to push for a new frontier and led up to a new horizon of performance achievement that cannot be achieved using conventional methods. PPMM has given birth to a new statistical signal processing tools. In this review, we will concentrate on the thermal PPMM methods have enabled spatial-, time-, frequency-, and sparse-patterns to be extracted according to the whole transient response behavior without resorting to any fixed library patterns. In such study, the physics principles and the signal processing models must be linked up, and understood prior to applying PPMM methods. The proposed method directly benefits the industry not only in terms of automatic defect detection but also enhances the detectability and resolution of the defects especially for such challenge tasks of detecting natural complex geometric cracks as well as micro defects. However, the advantages gained by the proposed methods will be augmented by the increase of computational power and higher data storage requirements of the processors. In our implementation, a high specification computer with a PC with of Core i7 32 GB RAM is required to implement all the proposed algorithms.

## Method

### Electromagnetic Thermal Conduction

When an electromagnetic (EM) field is applied to a conductive material, the temperature increases owing to resistive heating from the induced electric current which is known as Joule heating. Joule heating is the coupling of the electromagnetic and thermal fields. According to Joule’s Law:


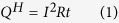


where *Q*^*H*^ is the sum of the generated heat, *I* represents electric current, *R* is electrical resistance, and *t* is time. According to the definition of electrical resistance:


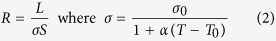


where *L* is the length of the conductor, *S* is the cross-sectional area of conductor, σ is the electrical conductivity which dependent on temperature *T* and *σ*_0_ is the conductivity at the reference temperature *T*_0_ and *α* is the temperature coefficient of resistivity, which describes how resistivity varies with temperature. Since the relationship between the current and the induced current density 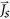 is:


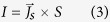


The Eqs (2) and (3) are substituted into Eq. (1) to obtain Eq. (4):


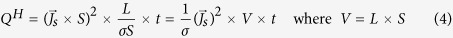


Consider the unit volume per unit time, thus Eq. (4) can be reduced to 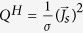. Following the energy conservation and Fourier heat conduction, the inductive heat conduction equation can be expressed as:


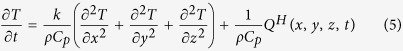


where *T* = *T*(*x*, *y*, *z*, *t*) is the temperature distribution, *k* is the thermal conductivity of the material (W/m K), which is dependent on temperature, *ρ* is the density (kg/m^3^), *C*_*p*_ is specific heat (J/kg K), and *Q*^*H*^(*x*, *y*, *z*, *t*) is the internal heat generation function per unit volume, which is the result of the eddy current excitation. The variation of temporal temperature depends on the spatial temperature variation for heat conduction. According to Eq. (5), heat conduction is influenced by *T*(*x*, *y*, *z*, *t*), *ξ*, *υ, σ, μ*, and *l*. Here *ξ* denotes the sensor geometry factor; *υ* denotes the parameter of the excitation (frequency, amplitude, etc.) and *l* denotes the lift-off (distance between the sensor and sample). From the above analysis, it becomes clear that the variation of temperature spatially and its transient response recorded from the IR camera directly reveals the intrinsic properties variation of the conductive material. In addition, variation of surface emissivity introduces spurious temperature inhomogeneity, according to Stefan-Boltzmann law^28^ the energy emitted by a black body per second per unit of area is proportional to the fourth power of its absolute temperature. This can be described as


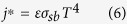


where *σ*_*sb*_ = 5.67 × 10 − 8W/(m2·k4) is the Stefan-Boltzmann constant and *T* is the absolute temperature. This difference can be described by the emissivity 0 ≤ *ε* ≤ 1 which denotes the ratio of radiation of actual object with respect to the black body. When the temperature of the material changes slightly, the radiation power will cause a large change. The radiance of the actual object depends on the properties of the material and the surface preparation besides the temperature.

### Interpretation of Electromagnetic Thermal Patterns

**Figure 1:**
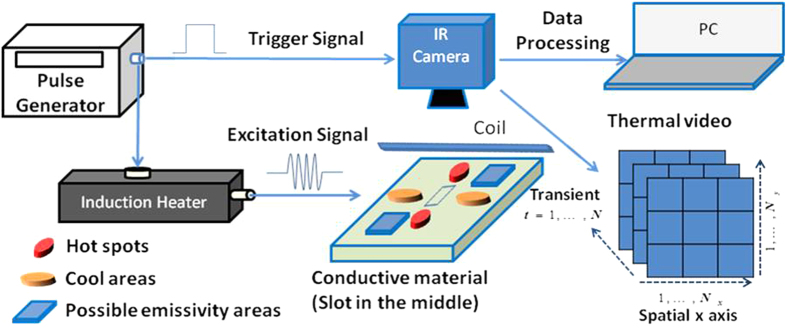
Electromagnetic thermal patterns. Electromagnetic Thermography and interpretation of thermal patterns.

Figure 1 shows the diagram of Electromagnetic Thermography NDT&E system. The excitation signal generated by the excitation module is a short period of high frequency current. It is driven to the coil on the conductive material. The current in the coil will subsequently induce eddy currents and generate the resistive heat in the conductive material. The heat will diffuse in time until it reaches an equilibrium state in the material. If a defect (e.g., crack) is present in the conductive material, eddy current distribution or the heat diffusion process will vary (as be interpreted in Fig. 1 bottom panel). Consequently, the spatial distribution of temperature on the surface of the material and the temperature transient response will show the variation. This is captured by an infrared camera as it records both the spatial and the transient response of temperature variation on the specimen. Mathematically, this can be represented as a spatial-transient tensor 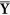 which has dimensions 
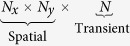
.

**Figure 2:**
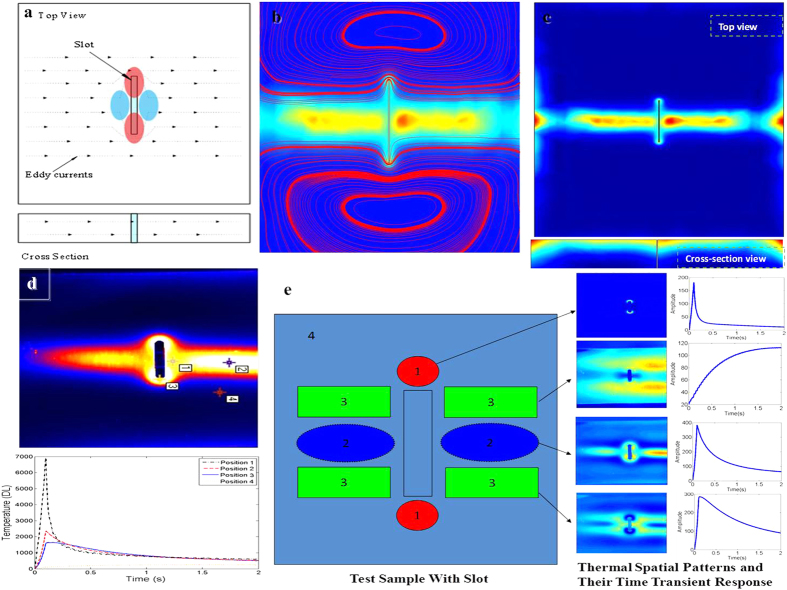
Illustration of Thermal- Electromagnetic patterns. (**a**) Schematic of theoretical EC distribution and resultant heating for slot. (**b**) Simulation of fused EC and heat distribution. (**c**) Simulation of heat. (**d**) Real test sample and its thermal spatial-transient signal. (**e**) Spatial-Transient pattern interpretation and separation results.

To interpret the thermal patterns, a sample with penetrated slot is used as example for one type of fundamental defect. Specifically, when eddy current encounters a discontinuity, e.g., a slot or notch, they are forced to divert, leading to areas of increased and decreased eddy current density. Therefore, in the heating phase, different areas have different heat generation rates which subsequently lead to temperature spatial variation. Hot spots are observed around the slot tips while the cool areas are located at both sides of the slot. In the cooling phase, heat diffuses from high temperature area to low temperature area, and reduces the contrast, the illustration can be seen in Fig. 2a. Figure 2b shows the numerical simulations for fusion of Eddy current and temperature distribution which performed using COMSOL Multi-physics simulation software via the electro-thermal module which combines the application mode for induction currents and general heat transfer. The heat transfer process and the magnetic field propagation were solved simultaneously by this module for an accurate description of the heating mechanism around a particular defect. The slot defect for the simulation are modeled in a steel block measuring 110 × 76 × 6.4 mm. The electrical and thermal parameters for the steel used in these simulations are shown in Table 1. A square coil with an outer diameter of 100 mm and inner diameter of 87.3 mm was used in the simulation. The coil is positioned perpendicular to the sample and at 90° to the defect. The current input for the coil was set at 350ARMS with an excitation frequency of 256 kHz.

Figure 2b illustrates the simulation results for the slot after 200 ms of heating. The eddy current (EC) flow and thermal distribution for the defect is visualized by the streamline plot in Fig. 2b,c. In the presence of a defect, EC will divert to complete their closed loop path which leaves a unique EC distribution based on the defect geometry that can provide useful multi-physics information to identify a defect. It illustrates the situation where EC cannot flow underneath the defect, hence are forced to flow around the end of the defect, leading to regions of high EC density and resulting in hot areas at the tips of the defect. The regions at either side of the defect are characterized with EC forced to spread out resulting in comparable cooler areas. The real test sample and its thermal spatial image after heating 0.1s and four positions of thermal transient curves are shown in Fig. 2d as well.

The streamline plots illustrate the defects with the geometry and orientation, but different positions on the sample under inspection can interact with the induced EC in different ways and cause characteristic heat spatial-transient distributions. The defect region is influenced by hybrid two physics of electromagnetic and thermal conduction and these mainly refer to factors of thermal conductivity *k* and generated heat due to eddy current density *Q*^*H*^(*x*, *y*, *z*, *t*). Thus, the thermal-transient pattern separation results (detailed description to be presented in next section) for slot tips (region 1 as shown in Fig. 2e) are seen with high induced current density and the temperature distribution according to Eq. (5) can be simply referred as 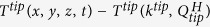, where *k*^*tip*^ is the thermal conductivity influenced by temperature of the defect (W/m K) and 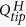 is the generated heat influenced by current density at slot tips. This happens as a result of high temperature rising rate generated in the heating phase. Fourier’s law of heat conduction states that the time rate of heat transfer through a material is proportional to the negative gradient in the temperature and to the cross section area of the material. Due to the singular areas around the slot tips, a high temperature gradient is generated at the end of heating phase. Therefore, a high falling rate is observed at the early stage of the cooling phase (transient curve of region 1 as shown in Fig. 2e). The cool area (region 2 as shown in Fig. 2e) with comparable lower induced current density, the temperature distribution can be referred as 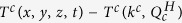 where *k*^*c*^ is the thermal conductivity influenced by temperature of defect (W/m K) and 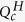 is the generated heat influenced by current density at side of slot. Compared to the area around crack tips, the area underneath the coil has a continual material distribution; compare to the area located at the side of the slots, the area underneath the coil has a higher eddy current density. Consequently, a moderate rising and cooling rate of transient in the heating phase and cooling phase are generated (transient curve of region 2 as shown in Fig. 2e). For non-directly heating region (region 4 as shown in Fig. 2e) with rare induced current density, the temperature distribution can be approximately considered as *T*^*NH*^(*x*, *y*, *z*, *t*) − *T*^*NH*^(*k*^*NH*^) where *k*^*NH*^ is the thermal conductivity influenced by temperature of material after induced heating procedure. As a result, the temperature rising rate in the heating phase is lower than the area underneath the coil, the slowest falling rate appears at region 4 while the temperature continuously rises at the beginning of the cooling phase (transient curve of region 4 as shown in Fig. 2e). For others, such as region 3 as shown in Fig. 2e, while high eddy current density appears around the slot tips, they are forced to spread out and result in lower density at either side of the slot. As a result, the temperature rising rate in the heating phase is lower than the area underneath the coil in which the slowest falling rate appears at region 3 while the temperature continually rises at the beginning of the cooling phase (transient curve of region 3 as shown in Fig. 2e). Therefore, the spatial-transient thermal distribution of tested sample *T* can be approximated as


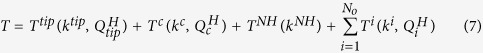


where 
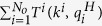
 denotes other possible characteristic thermal spatial-transient pattern region and *N*_*o*_ denotes the number. For example, the samples under test possibly have oil, oxide and other stains on the surface. Oil and oxide can drastically increase the thermal radiation, and results in their own characteristic spurious high temperature in the infrared images. Thus, all these characteristic thermal-transient regions can be considered as the pattern regions since each of them share similar transient responses in the sample. The infrared camera functions as an image capturing device which records the mixture contributed by each thermal pattern. To avoid the influences of arbitrary selection of image frame from the transient thermal videos, extraction of abnormal patterns has become ever more crucial. Therefore, the task of the proposed method is to separate the IR camera recorded image sequence into thermal pattern images as well as to automatically identify the one pattern image that relates directly to the defect. Specifically, **X**_*i*_(*t*), where *t* = 0, …, *N* are defined as thermal-transient patterns which have specific thermal region and characteristics in both spatial, transient and frequency domain. As an example, consider slot tips (region 1 as shown in Fig. 2e) with high induced current density, the temperature distribution according to Eq. (5) can be simply referred as 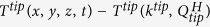. The Fourier’s law of heat conduction states that the rate of heat transfer through a material is proportional to the negative gradient in the temperature and to the cross section area of the material. For a uniform thickness plate used in this study, the cross section area is constant. Due to the singular areas around the slot tips, a high temperature gradient is generated at the end of heating phase. Therefore, a high falling rate is observed at the early stage of the cooling phase (transient curve of region 1 as shown in Fig. 2e). The thermal camera captures both the spatial and the transient response of temperature variation on the specimen which consist of thermal patterns **X**_1_(*t*), where *t* = 0, …, *N* that corresponds to 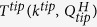. Similarly, Fig. 2e region 2 represents a cool area **X**_2_(*t*) with moderate rising and falling rate; region 3 represents a non-defect region **X**_3_(*t*) with high rising rate followed by a continually low speed rising and then drop down; and region 4 represents a non-excitation area **X**_4_(*t*) with continually temperature increasing. Mathematically, the thermography image captured by the infrared camera is considered as a mixing observation image **Y**(*t*). The term *m*_*i*_ is the mixing parameter which describes the contribution of the *i*^th^ position to the induced recorded thermography image in Fig. 1. In real applications, the number *N*_*s*_ is not necessary limited to 4 where *N*_*s*_ denotes the number of thermal patterns.

### Physics-based Modeling and Mining of Thermal Patterns in Spatial-Time, Frequency and Sparse domain

As there is only one infrared camera, this leads to the single channel signal separation problem. The mathematical model^29^ can be described as:


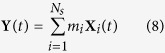


**Figure 3:**
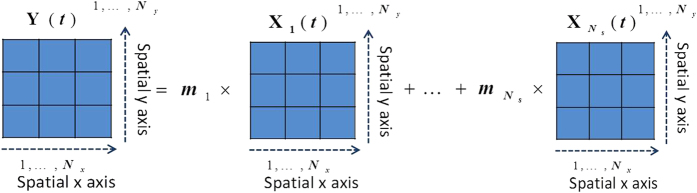
Mixing modeling. Mathematical representation of mixing process of ECPT.

The visual representation of Eq. (8) is shown in Fig. 3.

In Fig. 3, **Y**(*t*) and **X**_*i*_(*t*) denote the recorded image and the independent image generated by the area of position *i* at time *t* with dimensional *N*_*x*_ × *N*_*y*_, respectively. The task is automatically estimate **X**_*i*_(*t*) given only **Y**(*t*). To solve the above ill-posed problem, we adopt a decomposition-based approach The approach is used for analyzing non-stationary signals^30^ by expressing a fixed-length segment drawn from transient response, such that continuous transient slices of length *N* can be chopped out of a set of image sequences from *t* to *t* + *N* − 1, and the subsequent segment is denoted as equivalent as image sequences captured by *N* observations **Y′** = [vec(**Y**(*t*)), vec(**Y**(*t* + 1)), …, vec(**Y**(*t* + *N* − 1))]^**T**^ where ‘**T**’ denotes transpose operator and ‘vec’ denotes the vectorize operator. The constructed image sequences is then expressed as a linear combination of different thermal patterns such that


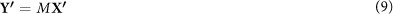


where 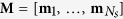 is the mixing matrix, **m**_*i*_ is the *i*^th^ mixing vector and 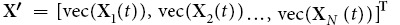. Assuming that *N*_*s*_ = *N* and **M** has full rank so that the transforms between **Y′** and **X′** be reversible in both directions such that we can find the inverse matrix **W** = **M**^−1^ which refers to the BSS method. The purpose of this decomposition is to model the multivariate distribution of **Y′** in a statistically efficient manner.

**Figure 4:**
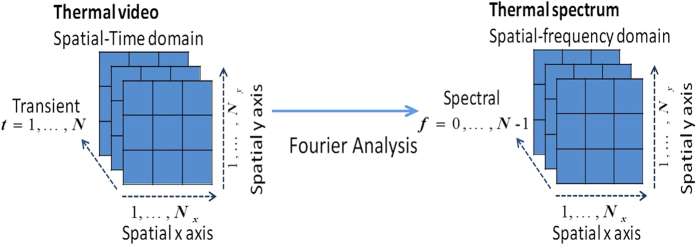
Time frequency transform. (**a**) Thermal spatial-transient domain and (**b**) Thermal spatial-frequency domain.

The phase information of thermography has been proved to offer deeper thickness of probing under the surface, less sensitivity to optical and infrared specimen surface features, better defect shape resolution, non-necessity to know a priori position of a non-defect area in the field of view to compute contrast image, ability to inspect high thermal conductivity specimens. In particular, the magnitude thermal image is proportional to local optical and infrared surface features. Of significant interest then is the phase image which being related to the propagation time delay, is independent of optical or surface features. Besides the spatial-time thermal pattern modeling, the spatial-frequency thermal pattern modeling is also investigated by computing the Discrete Fourier Transform (DFT) for each transient response:


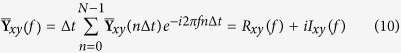


where, Δ*t* is the sampling time step, *R*(*f*) and *I*(*f*) are the real and imaginary components of 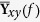, respectively. The subscript *x*, *y* are the coordinate index of spatial *x* and spatial *y*, respectively. This gives complex-valued tensor 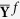 as shown in Fig. 4.

The spatial-phase spectrum from the pulse phase thermography system can be calculated as 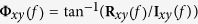 across the *x*- and *y*-axes. The observed spatial-phase spectrum is a sum of contribution from thermal events given by 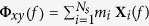 Similar to time domain analysis, thermography spatial-phase image **Φ**_*xy*_ is a 3-D tensor representation when taking in all (*x*, *y*, *f*) elements. Nonetheless, we can form a 2-D matrix format of the spatial-phase spectrum as 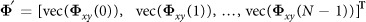. The aim of pattern separation is to factorize the spatial-phase matrix **Φ**^**'**^ into a product of two matrices as


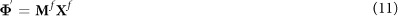


Not with standing above, the sparse factors enforce the solution to consider only the significant region where the defect may lie within the surrounding background. This is shown in Fig. 1. For data with sparse outliers or partially contaminated by noise of overwhelming magnitude, sheer low-rank assumption cannot fully capture its complex structure. Therefore, (9) and (11) can be considered as a model of combination of sparse pattern (e.g. hot spots) and non-sparse patterns


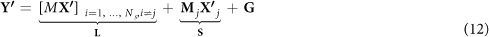


In reality, they play an important role in enhancing the defect detectability of IT system. A general assumption of Eq. (12) can be denoted as **Y′** = **L** + **S** + **G**, i.e., the pattern matrix **Y′** can be decomposed as the sum of a low-rank matrix **L** (e.g. for position 2, 3 and 4 reflected patterns in Fig. 1), a sparse pattern **S** (e.g. hot spots) which contains the spiky anomalies that are rarely shared by different instances, and **G** which is the noise term. When the algorithm optimizes the sparse patterns **S**, it finds the joint sparse estimation of **M**_*j*_ and **X′**_*j*_ (not just **X′**_*j*_). The sparse estimation of **X′**_*j*_ is required to obtain the correct shape of the pattern while the sparse estimation of **M**_*j*_ is to enable the user to determine the exact time when the target pattern takes place. In addition, the constraint of sparse factor *f*(**S**) can be selected optionally to satisfy different applications, where this could be selected as L_1_-norm (e.g.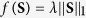 *λ* is sparse control parameter), L_2_-norm, Gaussian priors, Bernoulli sparse prior and so on. Similar to time domain analysis, thermography spatial-phase model can also be emphasized with sparse domain.

### Thermal Pattern Mining

**Figure 5:**
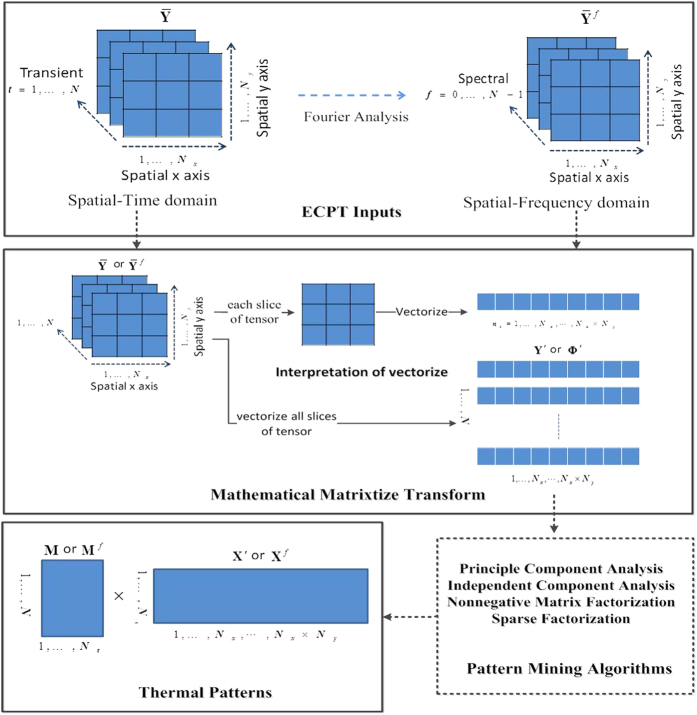
Procedure for thermal pattern mining. Schematic illustration of the spatial, time, frequency and sparse thermal pattern mining.

To avoid the influences of arbitrary selection of image frame from the transient thermal videos, the task of the pattern mining is to blind separate the observed **Y′** and **Φ**^**'**^ into different characteristic patterns **X′**, **X**^*f*^ and automatically identify the one which relates to defect. There exists several criteria for mining purpose such as principal component analysis which emphasize the decorrelation of separated patterns or independent component analysis where the separated patterns are said to be statistically independent^31^. The part-based patterns separation can be achieved by using nonnegative matrix factorization^32^ as well as sparse factorization^33^. The brief steps of pattern mining procedure have been summarized in Fig. 5.

## Results

**Figure 6:**
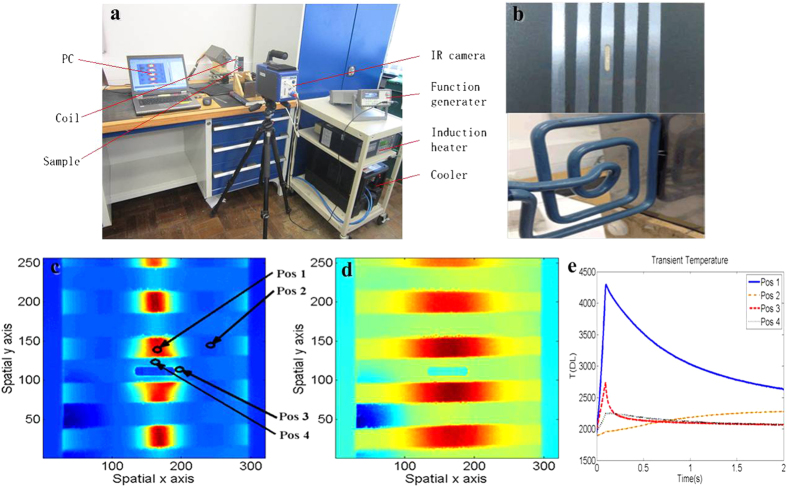
Experiment set up. (**a**) ECPT platform (**b**) Test sample (**c**) Original recorded infrared image at 0.1s (the end of heating phase) (**d**) Original recorded infrared image at 1.6s (the cooling phase) (**e**) Transient responses of different positions.

The experimental setup is shown in Fig. 6a. An Easyheat 224 from Cheltenham Induction Heating is used for coil excitation. The Easyheat has a maximum excitation power of 2.4 kW, a maximum current of 400 A_rms_ and an excitation frequency range of 150–400 kHz (380 A_rms_ and 256 kHz are used in this study). The system has a quoted rise time (from the start of the heating period to full power) of 5ms, which was verified experimentally. Water cooling of coil is implemented to counteract direct heating of the coil. The IR camera, SC7500 is a Stirling cooled camera with a 320 × 256 array of 1.5–5 μm InSb detectors. This camera has a sensitivity of <20 mK and a maximum full frame rate of 383 Hz, with the option to increase frame rate with windowing of the image. In this study, 2s videos are recorded in the experiments. A rectangular coil is constructed to apply directional excitation. This coil is made of 6.35 mm high conductivity hollow copper tube. A steel sample (0.24 × 45 × 100 mm^3^) with a slot of 10 mm length, 2mm width was prepared, as shown in Fig. 6a. Thermal conductivity of the stainless steel is 14 Wm^−1^K^−1^. There are equally spaced shinning and black stripes with 5 mm width on the sample surface. The bare strips are polished areas and the black strips were created by spraying the area with black paint to illustrate different emissivity (The emissivity of the black region is 1, which is the same for a blackbody. While, the emissivity of the shinning stainless steel surface is about 0.16). Therefore, the recording in thermal camera is the result of mixture of several complex thermal patterns (such as hot spots, cool area, emissivity area, non excitation area). This brings challenge task of the pattern mining methods. Thus, the validation of different automatic pattern mining methods is necessary. Of significant interest is the ability to accurately and automatically extract patterns that are directly related to defects. In the experiments, coil and IR camera were placed on the opposite side, presenting transmission mode. The coil was perpendicular to the slot and across the slot centre. Only one edge of the rectangular coil was used to stimulate eddy current in the sample, as shown in Fig. 6b.

**Figure 7:**
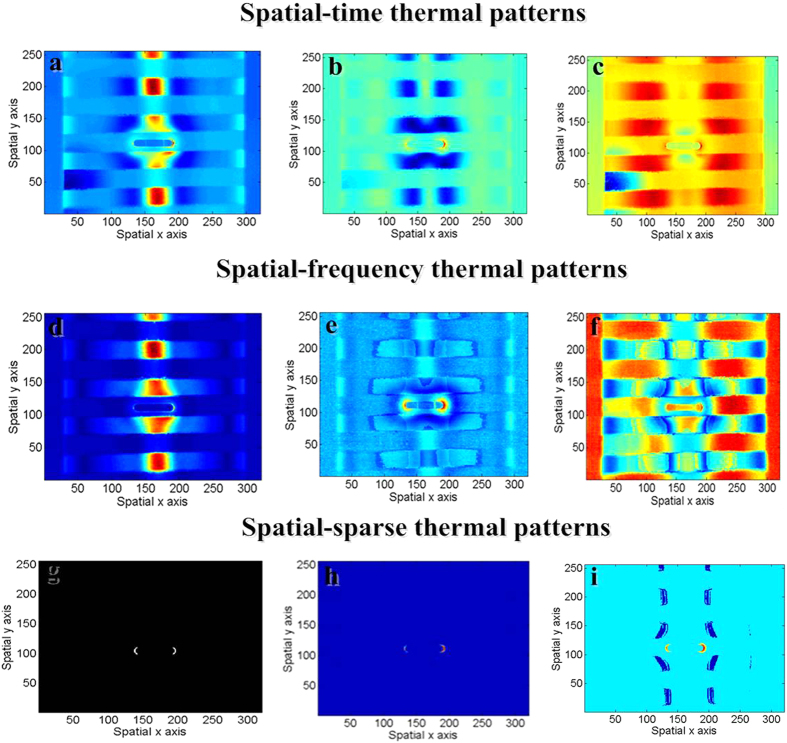
Thermal patterns. Mining thermal patterns using spatial-time domain pattern separation (**a–c**), spatial-frequency domain pattern separation (**d–g**) artificially annotation results of defect edgy, (**h**) sparse thermal pattern mining in spatial-time domain, (**i**) sparse thermal pattern mining in spatial-frequency domain.

Figure 6c shows the temperature distribution at the end of heating (0.1s). Due to the high emissivity of the black area, there is no obvious high temperature region around the slot tips. The high temperature can only be observed at the black area above the coil. In addition, Fig. 6d shows the temperature distribution at the cooling phase (1.6s). The high temperature still can only be observed at the black strip area because of both high emissivity and heat diffusion. The transient temperature behavior at different positions is shown in Fig. 4e. Pos 1 is at the crack side with black strip (high emissivity), Pos 2 is at the black strip where the area is far away from the excitation, Pos 3 is at the crack tip with the shinning strip, Pos 4 is at the crack side with shinning strip above the coil. As can be seen in Fig. 4e, different position behaves with different temperature transient characteristics. However, Pos 1 as well as other similar black strip area (above the coil) exhibits extreme high temperature transient in both heating and cooling phases in which other thermal patterns have been over-shadowed and therefore, they cannot be distinguished. Both transient and frequency domain are difficult to tackle in defect detection using the inductive thermography method where the hot spot around defect tips cannot be taken as an indicator of defects especially for small cracks. Notwithstanding this, it will lead to error when both black stain and cracks are present on the surface of the test sample. In order to solve this issue, the thermal pattern separation method is conducted. The thermal pattern separation methods are tested on recorded Thermal image sequences as described in Fig. 7. All methods are conducted using a PC with Intel Core i7 8GB RAM and the program platform is based on MATLAB. The experiments consist of three case studies: thermal-transient domain pattern mining, thermal-frequency domain pattern mining and thermal-sparse domain pattern mining.

Case (i): In order to validate the efficiency of spatial-time domain pattern mining methods, Principal Component Analysis (PCA), Independent Component Analysis (ICA) and Nonnegative Matrix Factorization (NMF) are tested to separate original thermal image sequences recorded by thermal camera. The number of initial thermal patterns *N*_*s*_ is varied as *N*_*s*_ = 2, 3, …, 10. Each method is validated by using Monte-Carlo repeated experiment involving more than 20 independent trials to get robust separation results. The optimal selection of method as well as *N*_*s*_ are based on subjective human judgement and cognitive control has been set in place to deal with any inter- judgement reliability issues. Finally, *N*_*s*_ = 3 and ICA are selected where three separated patterns directly indicating the specific thermal-transient characteristics. Figure 7a highlights the cool area with black strip (emissivity) where black region has spatial high emissivity and causes higher temperature. The transient characteristic of highlighted region pattern is similar to Pos 1 which can be seen in Fig. 6e. This pattern area underneath the coil has a continuous material distribution where the behavior of the transient response is independent of the emissivity. In order to prove this, Stefan-Boltzmann law states the energy emitted by a black body per second per unit of area is proportional to the fourth power of its absolute temperature. According to Eq. (6), the transient response of spatial pixel (*x*, *y*) can be presented as 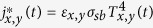 where *T*_*x,y*_(*t*) denotes the temperature response of the area corresponding to pixel (*x*, *y*), and is independent of the emissivity *ε*_*x,y*_. At the beginning of the heating phase, the sample is at the thermal equilibrium state *T*_*x,y*_(*t*_0_) = *T*_0_, *T*_0_ is equal to the ambient temperature. If the cooling time after this is long enough, the sample will reach a new thermal equilibrium state, *T*_*x,y*_(*t*_1_) = *T*_1_ where *t*_1_ denotes the elapsed time. Define 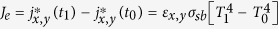 as the coefficient which is proportional to the surface emissivity. As the sample absorbs Joule heat generated by eddy current, *T*_1_ > *T*_0_, the transient response rise 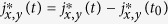 is divided by *J*_*e*_, namely


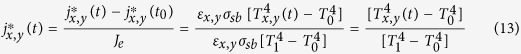


where 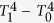 is the coefficient corresponding to heating power, time and the sample property, such as density, specific heat capacity, and quality. It is constant in each test. Therefore 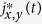 is independent of the emissivity, and characteristic of transient patterns does not be influenced by emissivity^37^. However, compared with separation, the time domain separated pattern of cool area still retains the mixing of hot spots around the right side of tips. Figure 7b highlights the hot spots where the transient characteristic of this thermal pattern is similar to Pos 3 as can be seen in Fig. 6e. Ideally, the extracted thermal-transient patterns should only highlight slot tips while the behavior of the transient response is the same as the curve with high rising and falling rate. However, the time domain separation recovers both sides of a hot spot but still mixes with stronger interference. Figure 7c highlights the region besides the excitation coil. The transient characteristic of this thermal pattern is similar to Pos 2 as can be seen in Fig. 6e. Ideally, the extracted thermal-transient patterns should only highlight non excitation region besides the coil and slot while the behavior of the transient response is the same as the curve which continuously rises. The separated results indicate that time domain separation does not completely separate this pattern and still mixes the patterns of both coil and slot.

Case (ii): In order to validate the efficiency of spatial-frequency domain pattern mining methods, similar to Case (i), PCA, ICA and NMF are tested and similar selection steps are conducted. Finally, *N*_*s*_ = 3 and ICA are selected. Figure 7d highlights the cool area with black strip (emissivity). Compared with the time domain separation, the thermal-phase pattern separation performs better since the interference interface from other source has been maximally suppressed. Similar better results are shown in Fig. 7e where thermal-phase pattern separation has fully recovered both sides of the hot spots and reduced the interference interface contrast. The reason is attributed to the feature of phase information that it is less sensitive to the optical and infrared specimen surface and better shape resolution. However, the extraction pattern of non excitation region besides the coil and slot has not been completely separated due to the interference interfaced from both coil and slot. This could be the reason that the phase image is highly related to the propagation time delay, is independent of optical or surface features.

Case (iii): In this case study both Thermal-sparse-spatial-time domain and Thermal-sparse-spatial-frequency domain pattern mining methods are conducted. The tested sparse pattern methods include: greedy sparse decomposition method^33^, variational Bayesian sparse decomposition with Gaussian sparse prior^34^ and Bayesian sparse decomposition based on Monte Carlo Markov Chain^35^. *N*_*s*_ = 2 is chosen since the sparse pattern separation only considers the pattern is either sparse or non-sparse. After test is conducted, the greedy sparse decomposition method is selected and more discussion will be found in the next section. In order to validate the detectability of sparse pattern, Fig. 7g shows the results of human annotation where the crack edge region (slot tips) is marked as “1” and the rest denoted as “0”. In Fig. 7h,i, by emphasizing the sparseness of solution, the edge of the hot spots on crack tips can be quantified and has benefitted the quantitative sizing of the defect. Both time and frequency domain spatial-sparse pattern mining methods has successfully accentuate the expected sparse locations which indicate the hot spots of the slot tips and suppressing the interference. The frequency domain method obtains better contrast between slot tips and others while it mixes interference from cool area besides the coil. On the other hand, the time domain method nearly completely extract the slot tips, however, the level of sparseness is not optimum where it loses information of the left side of tip. In order to quantitatively characterize the error rate of the separated pattern, the following section on Discussion will summarize the error rate of the proposed thermal pattern separation system.

## Discussion

In addition, it is found that emphasizing the sparseness of pattern extraction takes an important role in quantitatively analyzing the defect. In order to clarify this, the quantitative rate (QR)^36^ is introduced as


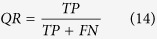


where *TP* refers to true positive which represent the situation where the sample contains a defect and the method indicates a defect is present as well as quantitative size of the defect, and *FN* refers to false negative which represents the situation where the sample does not contain a defect and the method does not indicate a defect is present as well as error quantitative size of the defect. The tested samples for validation study are set of steel samples (0.24 × 45 × 100 mm^3^) with variation slot size *L*_*j*_ × *W*_*j*_ where *L*_*j*_ = {10 mm, 9 mm, 8 mm} and *W*_*j*_ = {2 mm, 1.5 mm, 1 mm} width were prepared. The QR study is the comparison results between the referenced annotation with the pattern mining algorithms in spatial-time and spatial-frequency (phase) as well as sparse domain, respectively. The referenced human annotation defect region is shown in Fig. 7g. For Case (i) and (ii), only defect related thermal patterns are chosen for calculating QR, e.g. Fig. 7b,e.

**Figure 8:**
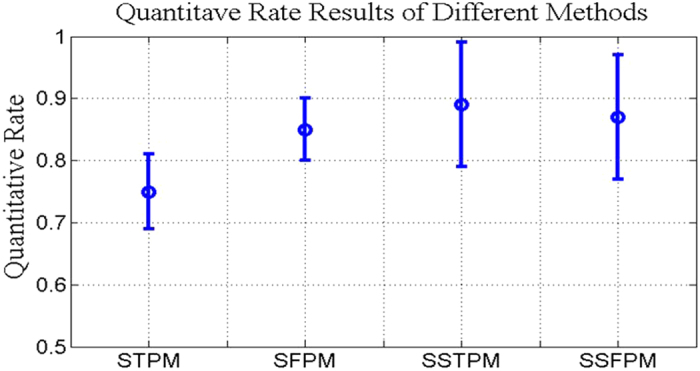
Comparison results. Quantitative rate comparison results of defect pattern mining by using different methods.

Figure 8 shows the quantitative rate comparison results for defect pattern analysis by using the different domain pattern mining methods with human annotation results: Spatial-Time Pattern Mining (STPM) with 0.75 ± 0.06, Spatial-Frequency Pattern Mining (SFPM) with 0.85 ± 0.05, Sparse Spatial-Time Pattern Mining (SSTPM) with 0.89 ± 0.1, Sparse Spatial-Frequency Pattern Mining (SSFPM) with 0.87 ± 0.1. The standard deviation is given by using Monte-Carlo repeated experiment involving more than 20 independent trials of different pattern mining algorithms such as PCA, ICA, NMF and *et al*. A higher performance is attained by emphasizing sparseness for both time and frequency domain pattern mining methods with an average accuracy of approximately 90%. However, the standard deviation varies significantly where the sparseness level is difficult to be controlled. The sparse pattern extraction method is highly reliant on *λ*. Thus to determine *λ*, model order selection is used where experiment is repeated by progressively increasing *λ* or using Bayesian strategy to estimate the *λ*. Although the update parameters has advantages to bypass human intervention, it brings the drawbacks of incorrect selection of prior distribution for the model parameters and results in convergence to local minima. This brings about sub-optimum performance.

In this paper, a thermal physics-based pattern modelling and mining based imaging methods have been proposed for NDT&E. The inductive thermography-based NDT methods have been tested to validate the method. The pattern modelling and mining methods allows thermal defects patterns to be extracted automatically for flaw contrast enhancement. The methods have been tested on man-made defects. Future work will focus on samples with complex surface condition, e.g. roughness and geometry variation. Complexity defects thermal pattern separation, e.g. subsurface defects, impact damage and delamination in composite structures will also be investigated.

## Additional Information

**How to cite this article**: Gao, B. *et al*. Electromagnetic Thermography Nondestructive Evaluation: Physics-based Modeling and Pattern Mining. *Sci. Rep*. **6**, 25480; doi: 10.1038/srep25480 (2016).

## Figures and Tables

**Table 1:** Electrical and thermal parameters setting.

Parameters	Steel
Conductivity, *σ* (S/m)	4.0319 × 10^6^
Relative permeability, *μ*	100
Temperature coefficient (K^−1^)	12.3 × 10^−6^
Density, *d* (kg/m^3^)	7850
Heat capacity, Cp (J/kg K)	475
Thermal conductivity, *k* (W/m K)	44.5
